# The Cyclooctadepsipeptide Anthelmintic Emodepside Differentially Modulates Nematode, Insect and Human Calcium-Activated Potassium (SLO) Channel Alpha Subunits

**DOI:** 10.1371/journal.pntd.0004062

**Published:** 2015-10-05

**Authors:** Anna Crisford, Ulrich Ebbinghaus-Kintscher, Eva Schoenhense, Achim Harder, Klaus Raming, Ita O’Kelly, Kelechi Ndukwe, Vincent O’Connor, Robert J. Walker, Lindy Holden-Dye

**Affiliations:** 1 Centre for Biological Sciences, University of Southampton, Southampton, United Kingdom; 2 Bayer CropScience Aktiengesellschaft, BCS AG-R&D-SMR-PC-PCB-PB, Monheim, Germany; 3 Bayer Animal Health GmbH, BHC-GDD-AH-PARA-AR, Monheim, Germany; 4 Faculty of Medicine, Southampton General Hospital, Southampton, United Kingdom; McGill University, CANADA

## Abstract

The anthelmintic emodepside paralyses adult filarial worms, via a mode of action distinct from previous anthelmintics and has recently garnered interest as a new treatment for onchocerciasis. Whole organism data suggest its anthelmintic action is underpinned by a selective activation of the nematode isoform of an evolutionary conserved Ca^2+^-activated K^+^ channel, SLO-1. To test this at the molecular level we compared the actions of emodepside at heterologously expressed SLO-1 alpha subunit orthologues from nematode (*Caenorhabditis elegans*), *Drosophila* melanogaster and human using whole cell voltage clamp. Intriguingly we found that emodepside modulated nematode (*Ce slo-1*), insect (*Drosophila*, *Dm slo*) and human (*hum kcnma1*)SLO channels but that there are discrete differences in the features of the modulation that are consistent with its anthelmintic efficacy. Nematode SLO-1 currents required 100 μM intracellular Ca^2+^ and were strongly facilitated by emodepside (100 nM; +73.0 ± 17.4%; n = 9; p<0.001). Drosophila Slo currents on the other hand were activated by emodepside (10 μM) in the presence of 52 nM Ca^2+^ but were inhibited in the presence of 290 nM Ca^2+^ and exhibited a characteristic loss of rectification. Human Slo required 300nM Ca^2+^ and emodepside transiently facilitated currents (100nM; +33.5 ± 9%; n = 8; p<0.05) followed by a sustained inhibition (-52.6 ± 9.8%; n = 8; p<0.001). This first cross phyla comparison of the actions of emodepside at nematode, insect and human channels provides new mechanistic insight into the compound’s complex modulation of SLO channels. Consistent with whole organism behavioural studies on *C*. *elegans*, it indicates its anthelmintic action derives from a strong activation of SLO current, not observed in the human channel. These data provide an important benchmark for the wider deployment of emodepside as an anthelmintic treatment.

## Author Summary

Filarial diseases affect an estimated 200 million people and the Drugs for Neglected Diseases initiative (DNDi) has identified development of macrofilaricidal drugs as a priority. Emodepside, currently used in companion animals, paralyses adult filarial worms and may address this unmet need for human medicine. Its receptor is an evolutionary conserved Ca^2+^-activated K^+^ channel, SLO-1. In this paper we address an important knowledge gap in terms of understanding the interaction of emodepside with its target receptor SLO-1 in nematodes in comparison to the human orthologue KCNMA1 and provide the first cross phyla analysis of the interaction of emodepside with slo channels, in nematode, insect and human. Intriguingly, this shows that emodepside modulates slo/BK currents from heterologously expressed channels from all three organisms, however there are discrete differences in the feature of modulation; only the nematode channel exhibits a sustained facilitation by emodepside. This is consistent with the effects of emodepside on *C*. *elegans* behaviour and indicates that this differential action of emodepside on the nematode channel likely underlies its potent anthelmintic effects. These data provide an important benchmark for the wider deployment of emodepside as an anthelmintic treatment.

## Introduction

Emodepside is a cyclooctadepsipeptide compound introduced into veterinary medicine for the treatment of nematode infections in companion animals [1,2]. It inhibits nematode development [3] and elicits profound impairment of neuromuscular function [4,5]. Recently there has been interest in the use of emodepside for the treatment of human helminthiases [6]. In particular its toxicity to adult filarial worms [7] has raised the prospect of it providing a much needed macrofilaricidal anthelmintic drug for the treatment of filarial diseases.

Investigation of the mode of action of emodepside revealed two targets, a latrophilin receptor [8,9] and a Ca^2+^-activated K^+^ channel SLO-1 [10] of which the latter appears to be the most important in terms of the efficacy of the compound at a whole organism level [10–13].

SLO-1 belongs to an evolutionary conserved family of K^+^ channels that are activated by cell depolarisation and cytosolic calcium. The first member of the family *slowpoke* (*slo*) was identified in the fruit fly *Drosophila melanogaster* [14] and the K^+^ channel was shown to have an unusually large conductance which prompted the name BK for ‘big K^+^ conductance’. slo/BK channels were subsequently found across the animal phyla, spanning nematodes to human [15]. Their dual regulation by membrane voltage and intracellular Ca^2+^ imparts a phylogenetically conserved role in regulating cell excitability (9).

SLO channels have been extensively studied in the model genetic organism, the nematode *Caenorhabditis elegans* [13,16,17] and it has provided an excellent experimental platform in which to investigate the mode of action [10] and selective toxicity [12,18] of emodepside. These studies have expressed slo/BK channels from parasitic nematodes and human in a *C*. *elegans slo-1* mutant to evaluate their role in conferring sensitivity to emodepside. These whole organism studies are consistent with the conclusion that emodepside preferentially activates the nematode isoforms of the calcium-activated K^+^ channel. More recently it has been shown that emodepside activates *C*. *elegans* SLO-1A heterologously expressed in *Xenopus* oocytes [19]. However a direct comparison of the effects of emodepside on heterologously expressed slo/BK channels from different phyla is currently lacking. This information is important to underpin its extended use as an anthelmintic drug.

To address this knowledge gap, here we provide the first cross phyla study on the actions of emodepside, on nematode, insect and human channels. We used heterologous expression of recombinant *C*. *elegans*, *Drosophila* and human BK/SLO channels to demonstrate that emodepside directly modulates the nematode, insect and mammalian channels. Importantly we provide mechanistic insight which reveals a differential action of emodepside on the nematode channel, a profound facilitation, which underpins its selective toxicity as an anthelmintic. Taken together our data further support the wider use of emodepside for the treatment of helminthiases. Moreover, they are consistent with BK/SLO channels harbouring a cyclooctadepsidpeptide pharmacophore which could be exploited for further applications in veterinary and human medicine.

## Methods

### Vectors for expression of slo/BK channels in HEK(Human Embryonic Kidney) 293 or CHO (Chinese Hamster Ovary) cells

*C*. *elegans slo-1a* (*Ce slo-1*) was cloned into a pIRES2-eGFP vector (BD biosciences, Clontech) for expression in HEK293 cells. *slo-1a* cDNA was PCR amplified from the pBK3.1 vector [16] and cloned into the pIRES2-eGFP vector.

In preliminary experiments we found that high concentrations of *pIRES2-eGFP*::*slo-1a* cDNA were required to obtain functional expression of SLO-1 currents in HEK293 cells. Due to simultaneous transcription of *egfp* this resulted in high levels of expression of eGFP and cells looked unhealthy (rounded, with dark inclusions) and were not suitable for reliable electrophysiology. Therefore, we removed the *eGFP* coding sequence from the *pIRES2-eGFP*::*slo-1a* vector and instead used a separate plasmid, at lower concentration, to transfect the cells with the *eGFP* transformation marker.

The mammalian orthologue for SLO-1a (NP_001024259), KCNMA1 was identified using Ensembl orthologue definitions as previously described [18]. It predicts 20 protein-coding transcripts. The protein product of Ensembl transcript KCNMA1-001 (peptide ENSP00000286627) corresponds to KCNMA1 variant 2/isoform b in the NCBI database (NP_002238). The alignment and identity of KCNMA1 isoform b with *C*. *elegans slo-1a* is described in Crisford et al, [18]The transcript encoding this isoform (NM_002247) was available to purchase from OriGene Technologies, USA. The *kcnma1* gene is in the pCMV6-XL4 vector behind a mammalian cell specific pCMV promoter suitable for expression in HEK293 cells.

*Drosophila slo* (*DmSlo*) was amplified by PCR with pfu Polymerase (Stratagene) from adult *Drosophila melanogaster* cDNA (Clontech) and cloned into pcDNA3.1 (Invitrogen).

### HEK293 cell culture

Human embryonic kidney cells (HEK293) were obtained from European Cell Culture, collection reference 85120602. Cells were maintained in 25cm^2^ flasks in Dulbecco's Modified Eagle Medium (DMEM GlutaMax, Gibco, Life Technologies UK) supplemented with 10% Fetal Calf Serum (FCS), 1% Penicillin/ Streptomycin (100units/100μg/ml) and L-glutamine (2 mM). Cells were incubated at 37°C, 5% CO_2_. HEK293 cells were passaged every 2–3 days (when 70–80% confluent) and kept for a maximum of 20 passages.

### HEK293 cell transfection

Plasmid DNA was delivered to the cells using jetPEI transfection reagent (Polyplus, Source Bioscience Autogen, Nottingham, UK). 1 g of DNA to express KCNMA1 or eGFP (KCNMA1 control) was mixed with 4 μl of jetPEI and each 1 μg of DNA to express SLO-1 or eGFP (SLO-1 control) was mixed with 2 μl of jetPEI. Transfections with eGFP DNA for control recordings were done in the same ratios as the transfections with the genes of interest. Cells were incubated with transfection mixtures for 18 hours at 37°C, 5% CO_2_. DNA/JetPEI complexes were then removed and replaced with fresh DMEM/10% FBS. Cells transfected with plasmid DNA to express SLO-1 were transferred to 30°C, 5% CO_2_ incubator for 24 hours as this enhanced heterologous expression of the [20] invertebrate channel in mammalian cells. Cells transfected with plasmid DNA to express KCNMA1 were maintained at 37°C.

### Generation of a stable *Drosophila melanogaster* Slo (Dm Slo) CHO cell line

CHO cell line (CHO duk) was obtained from ATCC, code ATCC CRL-9096. For transfection with plasmid DNA to express Dm Slo CHO cells were passaged to 40% confluence before adding the transfection solution. The transfection solution contained 300 μL OptiMEM (Life Technologies, Nr.: 31985), 2 μL (6μg) pcDNA3.1 (-)Dm Slo and 9μL FugeneHD (Promega, Nr.: E2311) and were added to the cells and incubated for 48 hours at 37°C, 5% CO_2_. The transfection medium was exchanged for the selection medium which contains additional G418 (2 mg/ml, Invitrogen, Nr.: 10131), the cells were seeded into 384 well plates (300 cells/well). After a few weeks, the few surviving cells were tested with a voltage sensitive dye (membrane potential assay kit Dye B, Molecular Devices Nr.: R80034) for K^+^ channel expression. Cells from the best clone (strongest signal in reaction to a 70 mM K^+^ application in combination with 3 μM Ca^2+^ Ionophor A23187, Sigma Nr.: C7522), were plated out on glass coverslips for testing Dm Slo channel expression electrophysiologically using whole cell voltage clamp technique (see section whole cell voltage clamping CHO). After validation of functional Dm Slo channel expression the best clone was subcloned in 384 well plates (0.7 cells/well) in order to obtain clonal purity. The clone with the best membrane potential signal was assessed electrophysiologically for Dm Slo channel function. This generated a final stable CHO cell line expressing the Dm Slo.

### Whole cell patch clamping HEK293 cells

Whole cell currents from cells transfected with plasmid DNA to express *Ce slo-1* were recorded 48 hours after transfection. Whole cell currents from cells transfected with plasmid DNA to express KCNMA1 were recorded 24 hours after transfection. Cells were transferred to the perfusion chamber and recordings were made at room temperature (20 to 25°C).

Patch pipettes (4–6 MΩ) were filled with internal solution containing 140 mM KCl, 1 mM MgCl_2_, 2 mM Na_2_ATP, 3.3 mM CaCl_2_, 10 mM Hepes, 5 mM EGTA, pH 7.2 (adjusted with KOH), in which free [Ca^2+^] was 300 nM. The calculation of free Ca^2+^ was made by using the software “MaxChelator Sliders”(from C. Patton, Stanford University) and a dissociation constant for the Ca^2+^-EGTA-complex of 90nM (pH 7.3) [21,22]. For 100 μM free [Ca^2+^] internal solution contained: 140 mM KCl, 1.2 mM MgCl_2_, 2 mM Na_2_ATP, 5.4 mM CaCl_2_, 10 mM Hepes, 5 mM EGTA. Osmolarity ranged from 280 to 300 mOsm. The external solution contained 137 mM NaCl, 5.9 mM KCl, 2.2 mM CaCl_2_, 1.2 mM MgCl_2_, 10 mM Hepes, 14 mM Glucose, pH 7.4 (adjusted with NaOH). Osmolarity was 300 mOsm.

Transiently transfected cells were identified by eGFP fluorescence. Whole cell currents were evoked by 50 ms voltage steps from -100 mV to +90 mV in 10 mV increments from a holding potential of -60 mV. Alternatively, whole cell currents were evoked by 50 ms voltage steps from -80 mV to +170 mV in 10 mV increments from a holding potential of -60 mV.

Cells were perfused with extracellular solution, with or without drug, at a rate of 0.5–0.8 ml min^-1^ using a parallel tubing perfusion system driven by an electric pump. The time taken for the drug to access the vicinity of the cell being recorded was 30 to 45 s from the initiation of perfusion. Depolarising voltage steps were applied from -60 mV to +70 mV for 100 ms cells were held at -60 mV for 10 s between depolarising steps in order to analyse the current-voltage relationship during drug applications. Drug or vehicle (control) application was initiated when the current elicited by the voltage steps from -60 mV to +70 mV reached a steady state (at least six current-voltage responses overlapped at +70 mV).

Whole cell currents were recorded using an Axopatch 200B amplifier (Axon Instruments, Foster City, CA) and pClamp 10 software employing Digidata 1440A (Axon Instruments). Data were filtered (4-pole Bessel) at 1 kHz and digitized at 5 kHz. Current-voltage relationships were plotted from the plateau of each depolarising step using Graph Pad Prism computer software (version 5.0 San Diego, California). The response to progressive depolarising voltage steps from -60 mV to a maximum of +170 mV was plotted as a time-dependent response. Leak subtraction voltage-command protocols were not applied. Traces for the whole cell currents where leak was more than 10% of the whole current were excluded from the analysis.

### Whole cell patch clamping CHO cells

Whole cell voltage-clamp was performed on CHO cells stably transfected with the Dm Slo channel. Patch pipettes (borosilicate glass capillaries, 2.5–3.5 MΩ) contained (in mM): 150 KCl, 10 HEPES (pH 7.3 adjusted with KOH), 10 K-EGTA, 0, 3, 5, or 7 mM CaCl_2_. The cells were placed in a perfusion chamber (2.5ml) at room temperature (22–25°C) and superfused continuously (flow rate 3 ml min^-1^) with external bath solution driven by gravity. The fluid in the chamber renewed every 60 s. The external bath contained (in mM): 150 NaCl, 4 KCl, 2 MgCl2, 2 CaCl2, 10 HEPES (pH 7.3 adjusted with NaOH). Compounds were applied to the cells using the U-tube reversed flow technique. Currents were recorded from a holding potential of -70 mV with an L/M-EPC 7 patch clamp amplifier (List, Darmstadt, Germany; pClamp software, Axon Instruments, Ver. 6.03, Foster City, CA), low-pass Bessel filtered at 3.15 kHz and digitized at 5 kHz sample rate.

### Ca^2+^ fluorescence measurements in HEK293 cells

HEK293 cells were incubated with 100 μl of 4 μM Fluo-3AM prepared with Pluronic F-27 (Molecular Probes, Life Technologies, UK) at room temperature for 30 min. Following dye removal cells were further incubated at 37°C, 5% CO_2_ with 180 μl of 1% Bovine Serum Albumin (BSA) per well for 30 min, washed with 200 μl of PBS (phosphate buffered saline) with subsequent addition of 100 μl of Hank’s Balanced Salt Solution (HBSS) (Gibco, Life Technologies, UK) containing 10 mM Ca^2+^. Ca^2+^ levels in cells before and after the treatment with emodepside or ionomycin were then quantified by measuring the Ca^2+^ fluorescence at excitation of 480p and emission of 525p using FLUO Star OPTIMA. The experiment was repeated at least three times with independent cell cultures and the data were pooled for analysis.

### Drugs

10 mM stock solution of emodepside was prepared freshly for each experiment in 100% DMSO (Dimethylsulfoxide). Emodepside was diluted in DMSO and then directly into external solution to give a final concentration of 100 nM, 10 nM and 1 nM (0.01% DMSO). For dissolving relatively high emodepside concentrations (1 μM and 10 μM) 0.03% Pluronic F68 (Sigma, P1300) was added as solvent. For injection studies emodepside was dissolved in 100% DMSO given a final concentration of 2 mg/ml. Emodepside was provided by Bayer Animal Health, Monheim Germany. 10 mM stock solution of penitrem A (Enzo Life Sciences, Exeter, UK) was prepared in 100% DMSO. Aliquots of stock solution were kept at -20°C. The final concentration of penitrem A was 1 μM (0.01% DMSO). Ionomycin was prepared by diluting 4 μl of ionomycin stock (10mM) in 100% DMSO into 36 μl of HBSS. 1 μl of this ionomycin solution was added to the microtitre cell in 100 μl of HBSS to give a final concentration of 1% DMSO and 10 μM ionomycin. 2% Triton-X 100 was prepared by diluting 10 μl of Triton-X in 490 μl of HBSS and 10 mM Ca^2+^ solution, 2.5 μl of this preparation was added per well (volume). For electrophysiological measurements a 1 mM stock solution of verrucologen (Cfm Oskar Tropitzsch e.K., Marktredwitz, Germany) was prepared in DMSO and diluted into external solution to give required concentrations between 0.1 pM and 30 μM just before the experiment.

### Statistical analysis

Data points in graphs are presented as the mean ± standard error of the mean for the number of experiments as shown in individual figures. Current-voltage and current-time relationships were plotted using either Graph Pad 5 software (San Diego, California) or Origin 6.0 Software (Microcal Software Inc., Northampton, MA, USA). Statistical significance was determined either by unpaired Student’s t-test, one-way or two-way ANOVA as appropriate; significance level set at P< 0.05, followed by Bonferroni post-hoc tests as appropriate. Boltzmann analysis was performed using GraphPad Prism, version 6.05 (San Diego, California) and the equation G/G_max_ = 1/(1+exp((V_50_-V_m_/slope)). Conductance, G, for each membrane potential (V_m_) was calculated using an equilibrium potential for K^+^ of -80 mV from the equation G = I/V_m_-E_K_. G_max_ was defined as the average maximal conductance for each experimental group. Values are given with 95% confidence intervals.

## Results

In this study we directly tested the hypothesis that emodepside is a modulator of Ca^2+^ -activated K^+^ channels in nematodes, insects and humans and characterised the features of modulation. First we established a heterologous expression assay for each of these channels and optimised the experimental conditions to record currents of similar amplitudes for the nematode, insect and human channel before progressing to investigate the effect of emodepside.

### Establishing conditions for heterologous expression of Ca^2+^ -activated K+ channels

**Fig 1:**
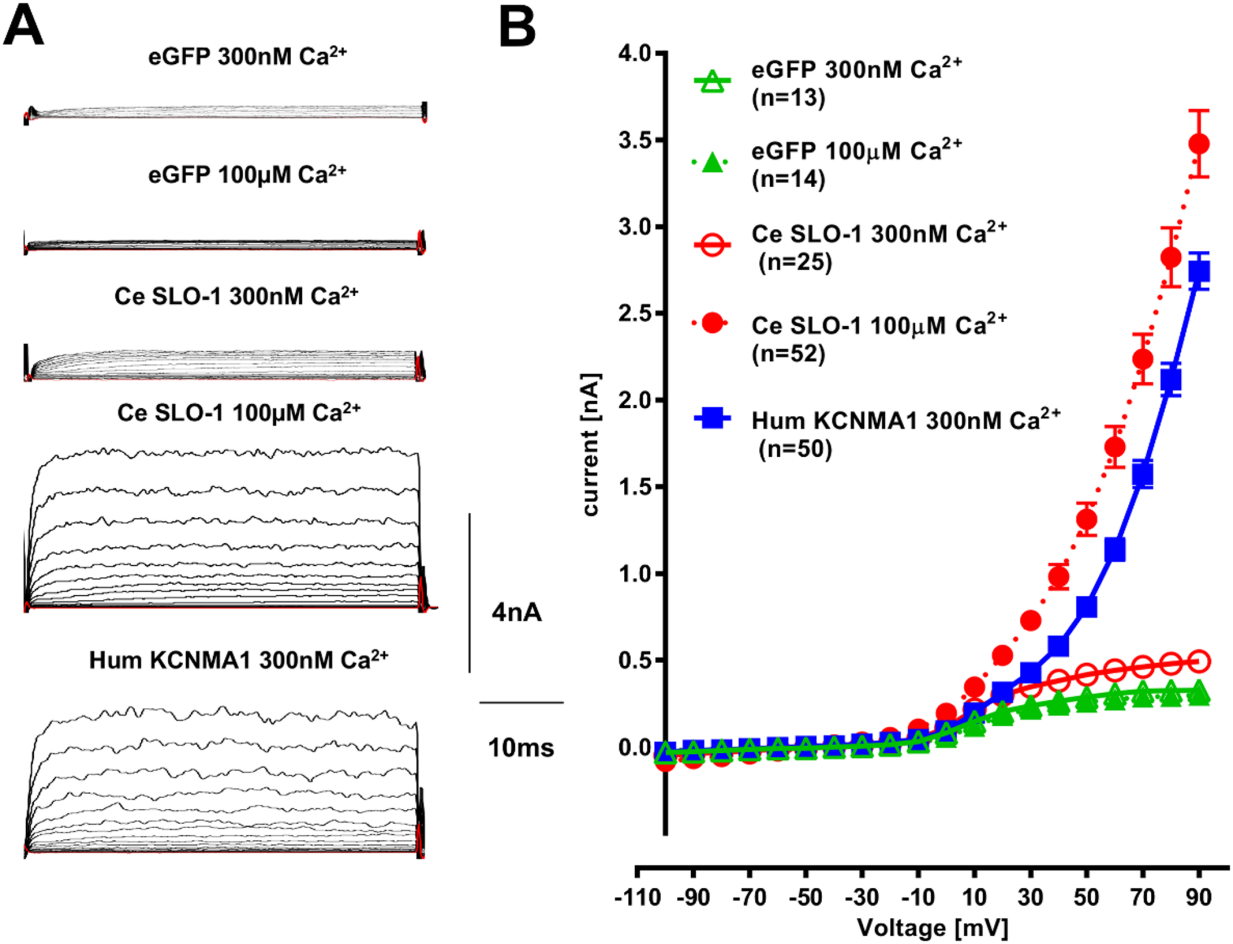
A comparison of the voltage and Ca^2+^ -dependent activation of human KCNMA1 and *C*. *elegans* SLO-1a. **A**. Representative traces of currents recorded from HEK293 cells expressing eGFP alone (control), Ce SLO-1 or hum KCNMA1. Membrane potential was held at -60mV and stepped to between -100 and +90mV in 10mV increments for 50ms. Free [Ca^2+^] in the internal solution was 300nM or 100μM as indicated. **B**. Current-voltage relationship for whole cell currents recorded from cells expressing eGFP alone (control), Ce SLO-1 or hum KCNMA1 at 300nM or 100μM free intracellular [Ca^2+^]. Membrane potential was held at -60mV and stepped to between -100 and +90mV in 10mV increments for 50ms. Data points are the mean ± s.e.mean of ‘n’ cell recordings (shown in brackets). Currents recorded from Ce SLO-1 in the presence of 300nM Ca^2+^, Ce SLO-1 in the presence of 100μM Ca^2+^ and hum KCNMA1 in the presence of 300nM Ca^2+^ were significantly different from eGFP recorded in 300nM and 100μM Ca^2+^; p<0.001, two-way ANOVA with Bonferroni post-hoc test.

**Fig 2:**
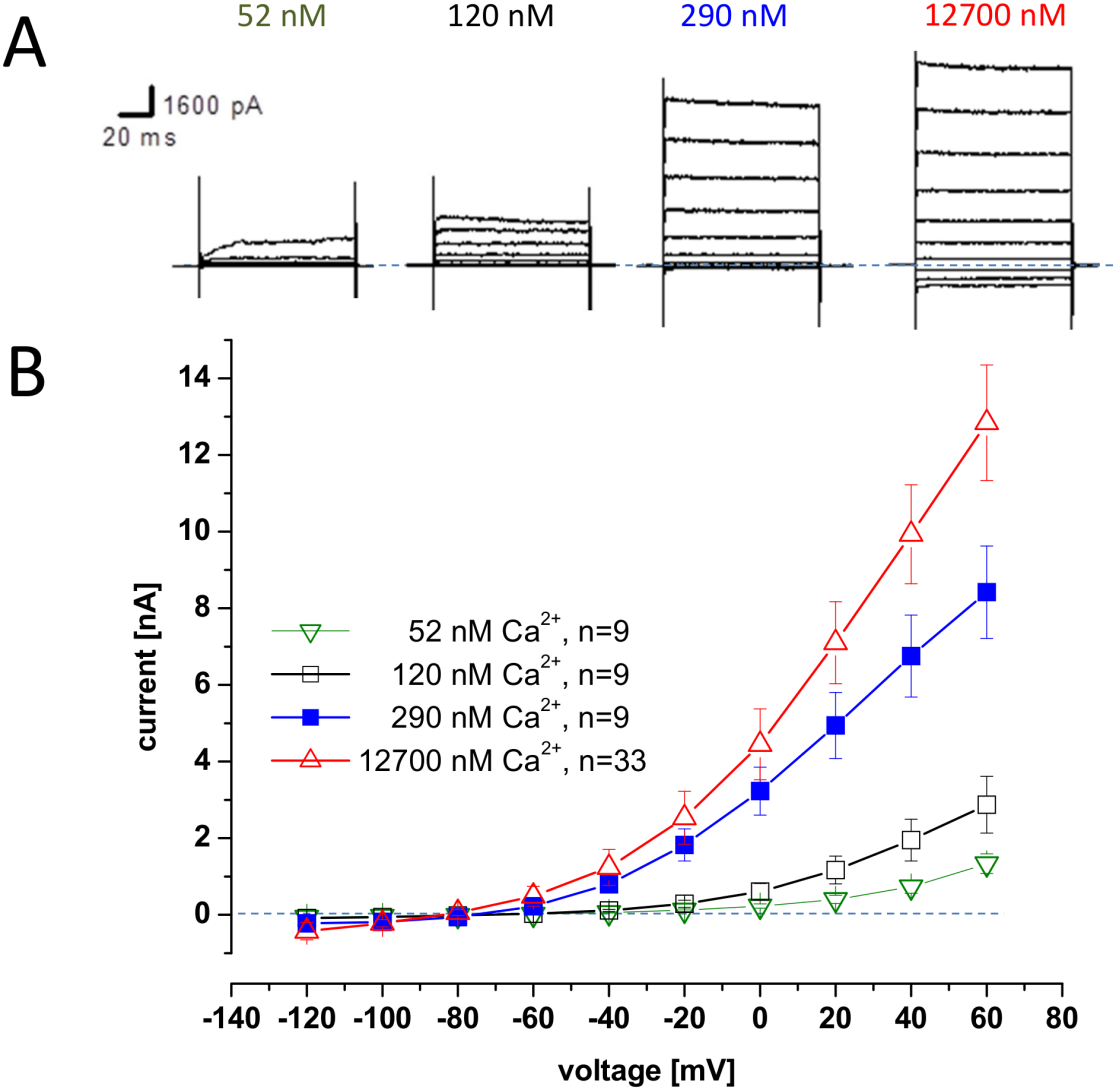
Voltage and Ca^2+^-dependent activation of Drosophila Dm Slo. **A**. Representative whole cell currents recorded from CHO cells stably expressing Dm Slo. Membrane potential was held at -70mV and stepped to between -120 and +60mV in 10mV increments for 100ms. Free [Ca^2+^] in internal solution was 52 nM, 120 nM, 290nM or 12700 nM. **B**. Current-voltage relationship of Dm Slo whole cell currents expressed in CHO at different free intracellular [Ca^2+^] as indicated. Membrane potential was held at -70mV and stepped to between -120 and +60mV in 10mV increments for 100ms. Data points are the mean ± s.e.mean of ‘n’ cell recordings, as indicated.

*Ce slo-1* (*C*. *elegans slo-1*) was expressed in HEK293 cells in the presence of different intracellular Ca^2+^ concentrations. Voltage-activated K^+^ currents of very low amplitude were recorded in 300 nM free intracellular Ca^2+^ (Fig 1A and 1B; mean peak current 0.49 ± 0.05 nA, n = 25) and were only marginally higher than the current recorded from control eGFP transfected cells (0.33 ± 0.03 nA; n = 13; P = 0.0192 unpaired Student’s t-test). Previously it has been reported that *C*. *elegans slo*-1 requires micromolar Ca^2+^ for activation [16,23,24]. Therefore, we tested the *slo-1* transfected cells in the presence of 100 μM Ca^2+^. Under this condition the voltage-activated K^+^ currents recorded from HEK293 cells were markedly increased (Fig 1A and 1B; mean peak current for SLO-1 in 100 μM Ca^2+^ was 3.48 ±0.19 nA; n = 52). Increasing the intracellular Ca^2+^ to 100μM had no significant effect on the currents recorded from cells expressing the control *egfp* plasmid (Fig 1A and 1B; mean peak current at +90 mV 0.30 ± 0.04 nA, n = 14; P = 0.57 unpaired Student’s t-test compared to *egfp* expressing cells recorded with 300 nM Ca^2+^) indicating that the effect of 100μM Ca^2+^ in facilitating channel activation is specific to the *slo-1* transfected cells. It is established that mammalian BK channels can be activated by Ca^2+^ with a Kd of 0.8–11 μM [25] and as low as 0.5–100 nM [26]. Consistent with this, and in contrast to the Ca^2+^ dependence observed for *slo-1* transfected cells, we observed robust voltage-activated K^+^ currents from cells expressing *kcnma1* with 300 nM intracellular Ca^2+^ (Fig 1A and 1B; mean peak current at +90mV of 2.74 ± 0.11nA, n = 50; P<0.001 compared to *egfp* alone, unpaired Student’s t-test). Similarly CHO cells expressing *Dm Slo* (*Drosophila melanogaster Slo*) showed strong activation of voltage-activated K^+^ currents at low free intracellular Ca^2+^ (Fig 2A and 2B) with a threshold around 52 nM and robust activation at 290 nM. Therefore, in further experiments to characterise all three channels we routinely used 100 μM, 300 nM and 290 nM free intracellular Ca^2+^ for *Ce slo-1*, human *kcnma1* and *Dm Slo* respectively in order to obtain robust currents of similar amplitude for each of the channels against which to make the most accurate comparison of the effects of emodepside on the different isoforms of channel.

**Fig 3:**
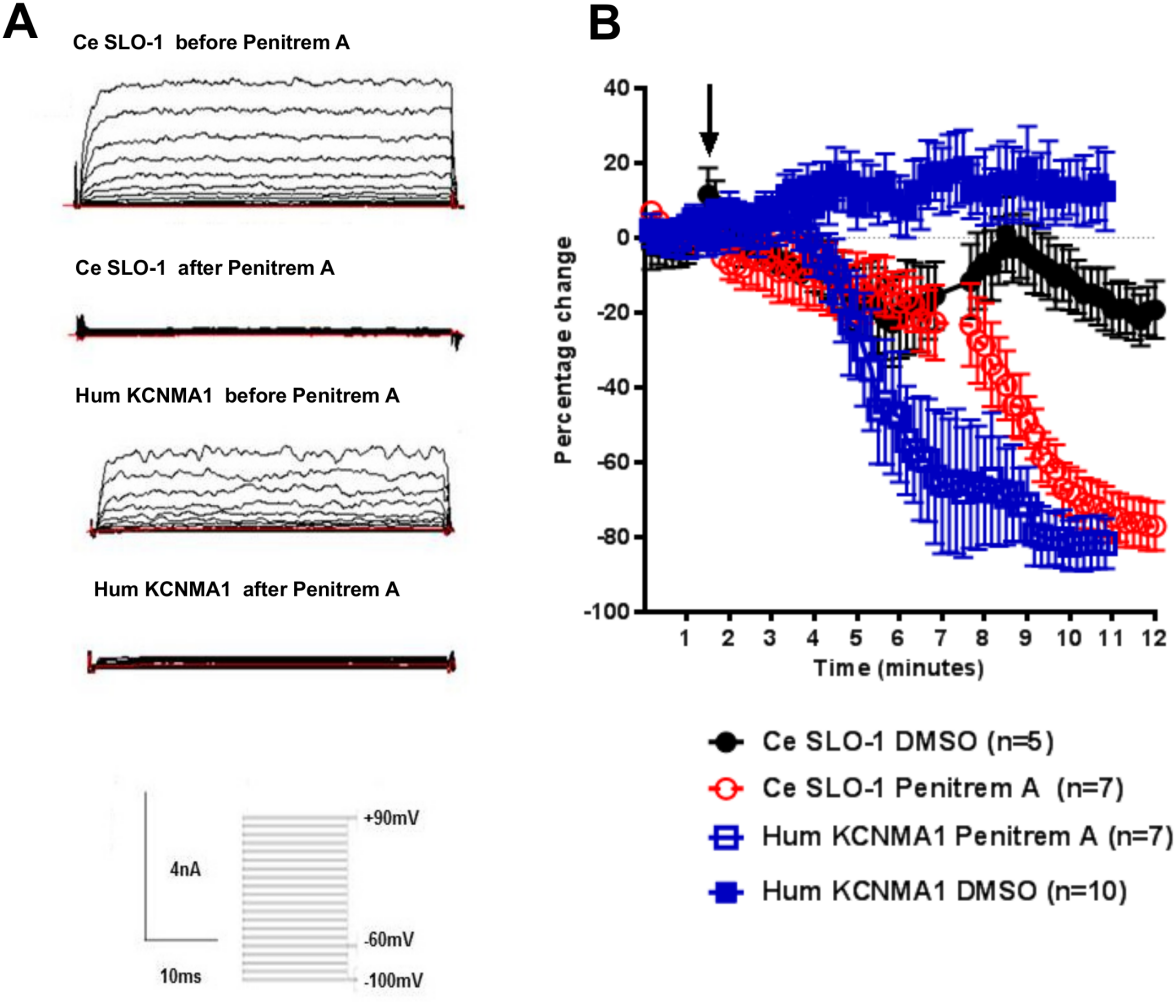
Ce SLO-1 and hum KCNMA1 currents are blocked by the BK channel antagonist penitrem A. **A**. Representative whole cell currents recorded from HEK293 cells expressing either Ce SLO-1 or hum KCNMA1 before and after penitrem A (1μM) application. Membrane potential was held at -60mV and stepped to between -100 and +90mV in 10mV increments for 50ms. **B**. A time-course of steady state Ce SLO-1 and hum KCNMA1 currents at +70mV (stepped every 10s for 40ms from -60mV holding potential) before and during application of DMSO (0.01%) or penitrem A (1μM). The current response is shown as a percentage change from the mean of pre-drug current amplitude during the 0 to 2 min time period. An arrow indicates the time point at which the drug solution reached the chamber with the cells. Free intracellular [Ca^2+^] was 100μM for Ce SLO-1 and 300nM for hum KCNMA1. Data points are the mean ± s.e.mean. p<0.0001 for penitrem A inhibited current compared to DMSO treatment, two-way ANOVA with Bonferroni post-hoc tests.

**Fig 4:**
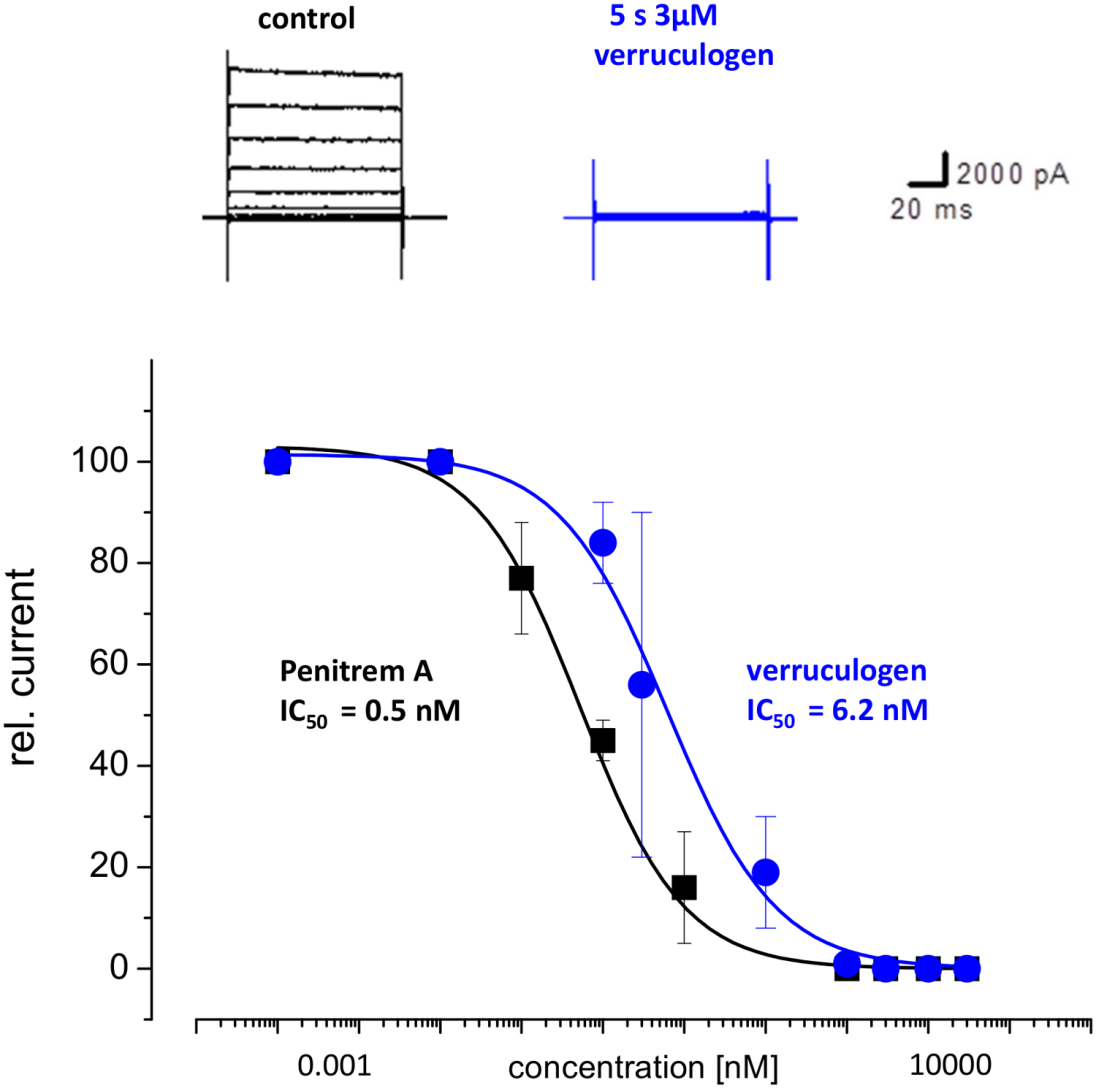
Dm Slo currents are blocked by the BK channel antagonists verruculogen and penitrem A. Top panel shows representative traces of Dm Slo whole cell currents stable expressed in CHO cells before and after verruculogen (3μM) application. Membrane potential was held at -70mV and stepped to between -120 and +60mV in 10mV increments for 100ms. Bottom panel shows dose-response curves for verrucologen (circles) and penitrem A (squares) on Dm Slo. Membrane potential was held at -70mV. Test pulses to + 60mV for 100ms. Each data points represent the mean ± s.e. of n = 3 cells.

We confirmed the identity of the *Ce slo-1*, *kcnma1* and *Dm Slo* channel currents by testing their sensitivity to selective BK channel blockers penitrem A [27] or verruculogen [28](Fig 3A and 3B; Fig 4). Only cells for which a consistent current-voltage relationship was obtained for the first 2 min of recording were subjected to drug application. For *C*. *elegans* SLO-1 in which recordings were made with 100 μM intracellular Ca^2+^ such stable recordings were only achieved for 10% of the total number of transfected cells from which whole cell recordings were made, most likely reflecting the impact that the high Ca^2+^ concentration has on the integrity of the cells. Once a stable recording was established the drugs were applied and recordings were made throughout the drug application for a period of up to 15 min. This was the maximum duration of recording that could be made for SLO-1 as after this time the recordings became unstable as indicated by the holding current and the cells were routinely lost. Visual inspection of the cells indicated a shrunken appearance, presumably due to the high (100 μM) intracellular Ca^2+^ that was required for these recordings. For SLO-1, the recordings were interleaved with controls on the same day; control recordings were made from transfected cells that were perfused with vehicle control (0.01% DMSO). Both SLO-1 and KCNMA1 currents were blocked by penitrem A (1 μM; Fig 3A and 3B). The mean peak current at +90 mV for SLO-1 was 3.95 ± 0.73 nA before and 0.63 ± 0.17 nA after 10 min of treatment with penitrem A (n = 7). For KCNMA1 the mean peak current at +90 mV was 2.66 ± 0.20nA before and 0.53 ± 0.09 nA after 10 min of treatment with penitrem A (n = 7). Similarly, in CHO cells expressing *Dm Slo* the voltage-activated K^+^ currents elicited in the presence of 290 nM free intracellular Ca^2+^ were completely blocked by the BK channel antagonist verruculogen at 3 μM (Fig 4; n = 5) and the dose response curve revealed an IC_50_ value of 6.2 nM for inhibition of Dm Slo by verruculogen (95% confidence intervals 2.52 to 8.57 nM) and an IC_50_ value of 0.5 nM for inhibition of Dm Slo by penitrem A (95% confidence intervals 0.43 t0 12.9 nM; Fig 4).

### Biphasic modulation of human KCNMA1 by emodepside

**Fig 5:**
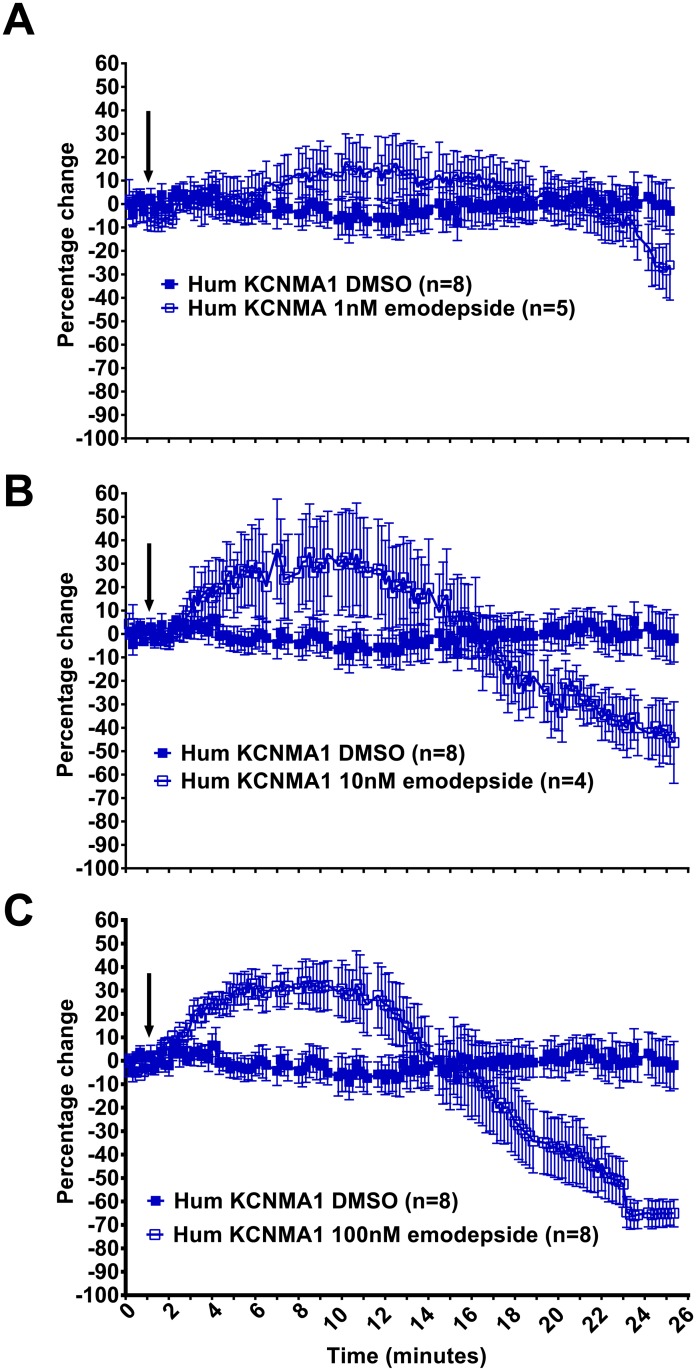
The biphasic effect of emodepside on hum KCNMA1 currents. Time-course analysis of steady state hum KCNMA1 currents expressed in HEK293 cells at +70mV (stepped every 10s for 40ms from -60mV holding potential) before and during application of 0.01% DMSO or emodepside. The current response is shown as a percentage change from the mean of pre-drug current amplitude during the 0 to 2 min time period. Arrow indicates the time of emodepside application at **A**. 1nM **B**. 10nM and **C**. 100nM. Data points are the mean ± s.e.mean. 100nM emodepside had a biphasic effect on hum KCNMA1 currents eliciting a significant increase between 4 and 8 min application (p<0.05) and a significant decrease after 20 min application (p<0.001); two-way ANOVA with Bonferroni post-hoc tests.

The same protocol that was optimised to determine the penitrem A inhibition of KCNMA1 was modified to test for an interaction of emodepside with the channels. Thus, for these experiments emodepside (1, 10 or 100 nM) or DMSO (0.01%) was applied to the cells after 2 min initial baseline recording and recordings were followed for up to a further 23 min i.e. as long as the stability of the recording permitted. This range of concentrations of emodepside was chosen as the EC_50_ for the inhibitory effect of emodepside on *C*. *elegans* behaviours is between 10 and 100 nM [10,18]. In HEK293 cells expressing KCNMA1 emodepside elicited a biphasic, concentration-dependent effect on current amplitude (Fig 5A, 5B and 5C). The effect was of a similar magnitude for 10 nM and 100 nM emodepside but was only statistically significant for 100 nM emodepside. The early facilitation was transient, lasting less than ten min, and followed by an inhibition of current amplitude. Again the inhibition was observed at both 10 and 100 nM emodepside but was only statistically significant for 100 nM emodepside. The mean peak currents for KCNMA1 at +90 mV were increased from 2.94 ± 0.36 nA (n = 8) to 4.31 ± 0.34 nA (n = 8) after 5 min emodepside and were then reduced to 1.67 ± 0.26 nA (n = 7) after 23 min of emodepside. Whole cell currents recorded from transfected cells treated with 0.01% DMSO did not change significantly over a 25 min time-course. The time-course of the response to emodepside was slow in onset and slow to reach the peak effect similar to the time-course of the effect of emodepside reported in other systems [13].

### Facilitation of *C*. *elegans* SLO-1 by emodepside

**Fig 6:**
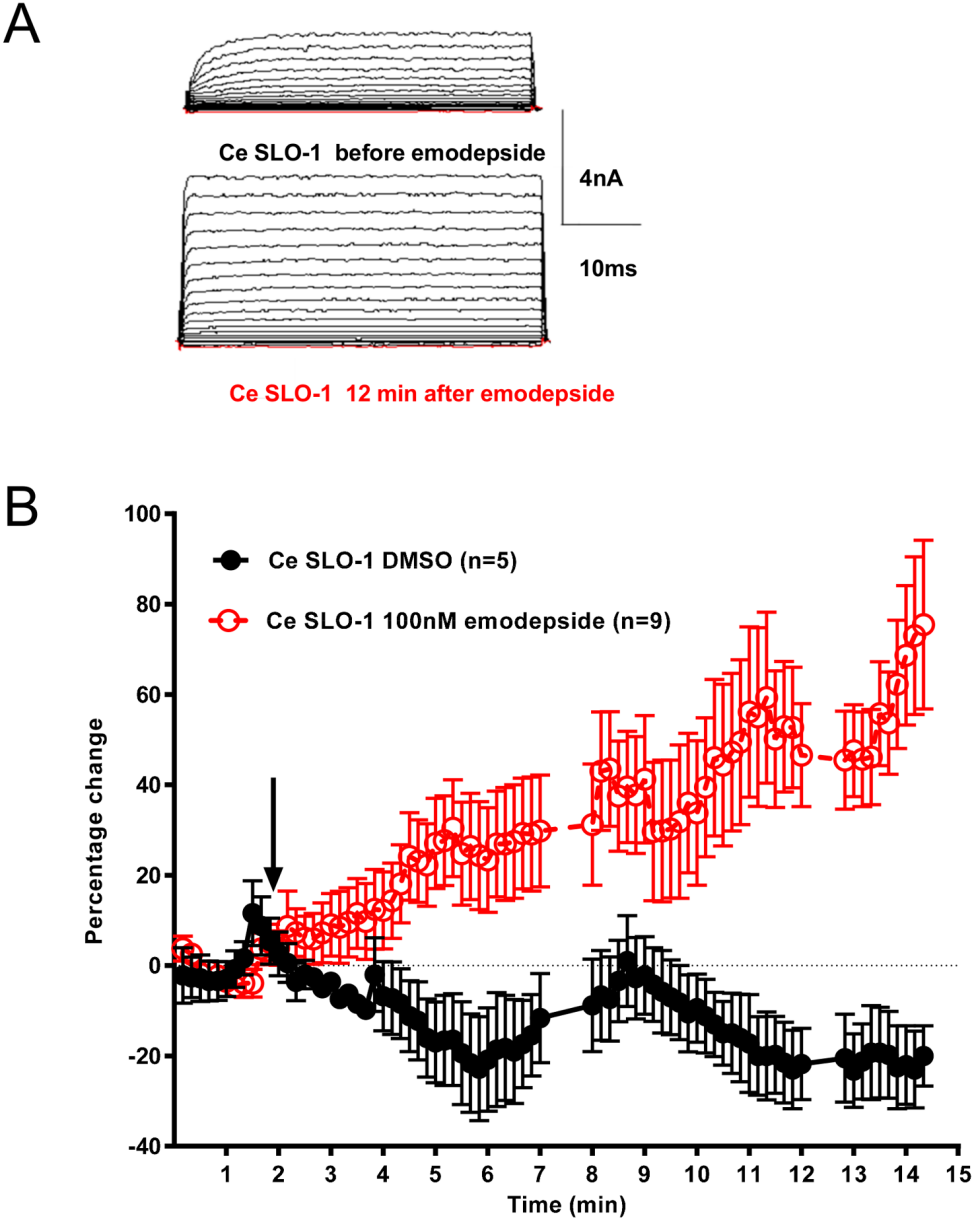
Emodepside increases Ce SLO-1 currents. **A**. Representative traces of Ce SLO-1 whole cell currents expressed in HEK293 cells before (top trace) and (bottom trace) 12 min after emodepside (100nM) application. Membrane potential was held at -60mV and stepped to between -100 and +90mV in 10mV increments for 50ms. **B**. A time-course of steady state Ce SLO-1 currents expressed in HEK293 cells at +70mV (stepped every 10s for 40ms from -60mV holding potential) before and during application of 0.01% DMSO or 100nM emodepside. The current response is shown as a percentage change from the mean of pre-drug current amplitude during the 0 to 2 min time period. Arrow indicates the time of emodepside application. Data points are the mean ± s.e.mean. Emodepside significantly increased the amplitude of the Ce SLO-1 currents, p<0.001, two-way ANOVA with Bonferroni post-hoc tests.

Given the effect of 100 nM emodepside on hum KCNMA1 (huma KCNMA1) we decided to test the effect of this concentration on *C*. *elegans* SLO-1 currents. We were constrained to testing this concentration of emodepside rather than a dose range because of the technical difficulty of recording SLO-1 currents from HEK293 cells with 100 μM Ca^2+^. Notably, for Ce SLO-1 the current amplitude was increased, but not inhibited, by 100 nM emodepside (Fig 6A and 6B). The facilitation followed a slow time-course and continued to increase throughout the time-course of the experiment (Fig 6B). The mechanisms that might underpin a slow time-course of action for emodepside, pharmacokinetic versus mechanistic, have previously been discussed in Holden-Dye et al [13]. The mean peak currents at +90 mV for Ce SLO-1 recordings were 2.95 ± 0.35nA before and 4.39 ± 0.55nA after 10 min of treatment with emodepside (n = 9; Fig 6B) representing a facilitation of 73.0 ± 17.4% (n = 9; p<0.001 compared to DMSO control). Over the same time-course the 0.01% DMSO control treated transfected cells exhibited mean peak currents at +90 mV of 3.14 ± 0.36nA before DMSO compared to 2.17 ± 0.25nA after DMSO (n = 5).

### Emodepside shifts the voltage activation curve for Ca^2+^ -activated K^+^ channels

**Fig 7:**
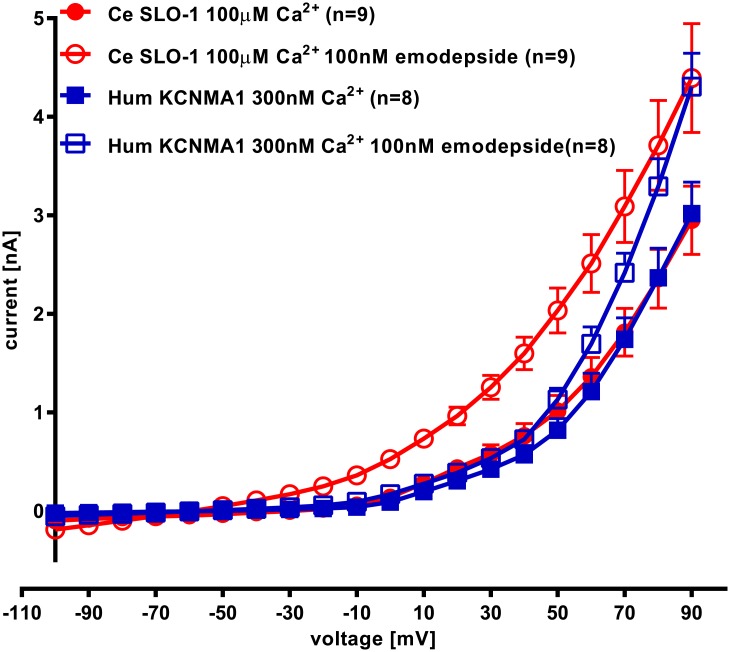
The effect of emodepside on voltage-activation of Ce SLO-1 and hum KCNMA1. Analysis of the current-voltage relationship of the data sets shown in Fig 6 and Fig 7 for Ce SLO-1 and hum KCNMA1 whole cell currents expressed in HEK293 cells before and after treatment with emodepside (100nM). Ce SLO-1 currents were recorded before and 12 min after emodepside application. Hum KCNMA1 currents were recorded before and 5 min after emodepside application. Membrane potential was held at -60mV and stepped to between -100 and +90mV in 10mV increments for 50ms. Data points are the mean ± s.e.mean of n = 9 for Ce SLO-1 and n = 8 for hum KCNMA1. The amplitude of Ce SLO-1 currents in the presence of 100nM emodepside was significantly greater than currents recorded in the absence of emodepside between +20 and +90mV and for hum KCNMA1 was significantly greater than currents recorded in the absence of emodepside between +70 and +90mV; p<0.05; two-way ANOVA with Bonferroni post-hoc tests.

The voltage activation of Ce SLO-1 and hum KCNMA1 was tested before and during the peak of the emodepside facilitation (i.e. after 12 min application of 100 nM emodepside for Ce SLO-1 and after 5 min application of 100nM emodepside for hum KCNMA1; Fig 7). A significant shift in the voltage activation curve was observed for Ce SLO-1 (p<0.05) after emodepside treatment and a smaller significant shift for hum KCNMA1 (p<0.05). The data from Fig 7 were subjected to Boltzmann analysis to estimate V_50_, the membrane potential for half-activation of the channels using G_max_ values of 0.0174, 0.0258, 0.0177 and 0.0253 μS for Ce SLO-1 control, Ce SLO-1 with emodepside, hum KCNMA1 control and hum KCNMA1 with emodepside, respectively. For Ce SLO-1 V_50_ was +49.74 mV (95% confidence intervals 45.46 to 54.01; V slope 20.66) and this was shifted in the presence of emodepside to +32.95 mV (95% confidence intervals 28.82 to 37.07; Vslope 28.36). For hum KCNMA1 the V_50_ was +56.51 mV (95% confidence intervals 52.82 to 60.20; Vslope 17.45) which was unchanged in the presence of emodepside, + 57.88 mV (95% confidence intervals 54.09 to 61.68; Vslope 17.12). This analysis confirms the selective effect of emodepside on the voltage-activation for Ce SLO-1.

### The effects of emodepside on Dm Slo

**Fig 8:**
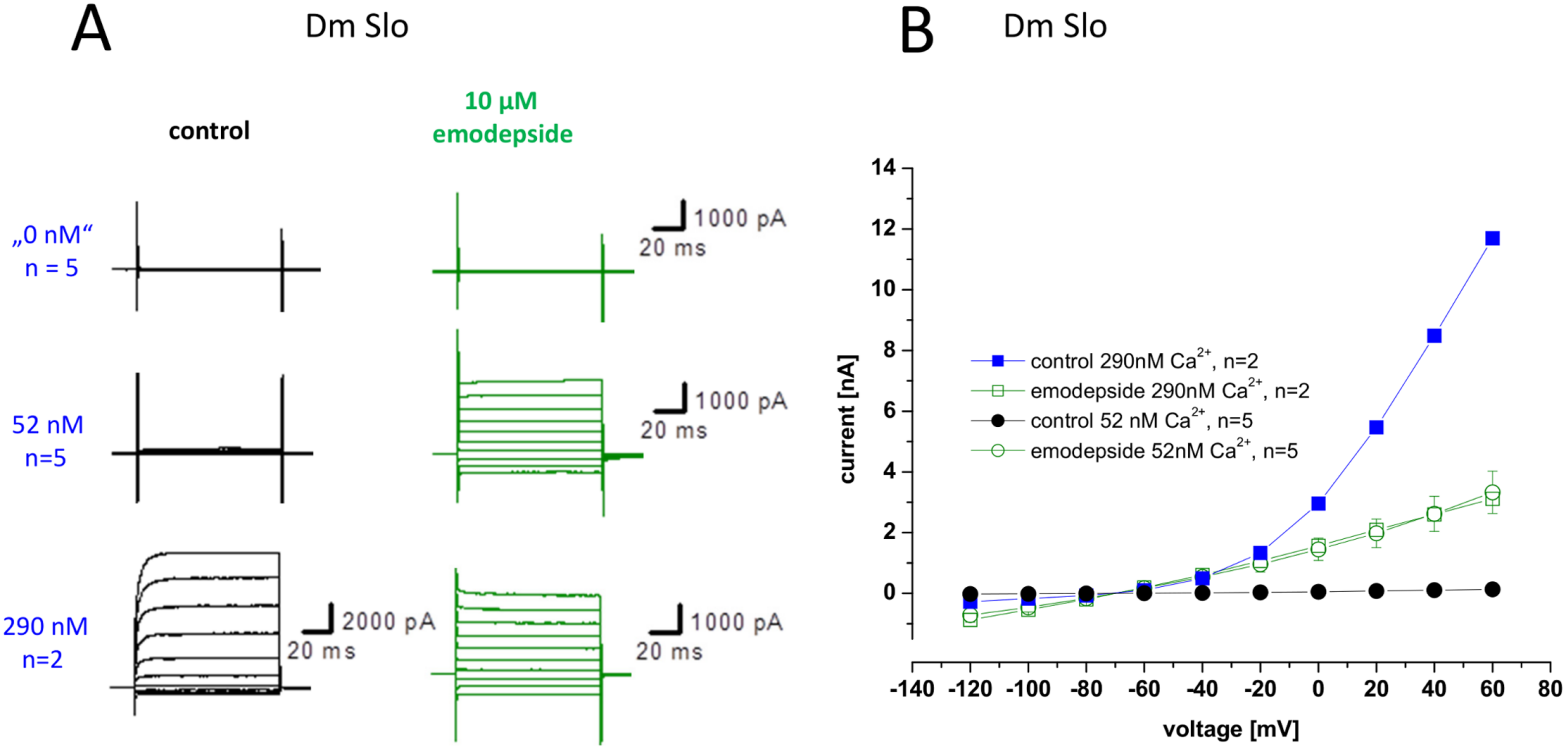
Intracellular Ca^2+^ ions are required for emodepside activation of Dm Slo. **A**. Representative traces of Dm Slo whole cell currents stable expressed in CHO cells before (left hand traces) and after (right hand traces) emodepside (10 μM) application at different free intracellular [Ca^2+^] (“0 nM”, 52 nM and 290 nM as indicated). Emodepside was applied for 10 min for experiments with low free intracellular [Ca^2+^] (“0 nM” and 52 nM). Membrane potential was held at -70 mV and stepped to between -120 and +60 mV in 10 mV increments for 100 ms. **B**. Current-voltage relationship of Dm Slo whole cell currents after emodepside application at two different free intracellular Ca^2+^ concentrations, as indicated. Membrane potential was held at -70 mV and stepped to between -120 and +60 mV in 10 mV increments for 100 ms. Data points are the mean ± s.e.mean of n cell recordings.

Recordings made from CHO cells expressing *Dm Slo* revealed complex effects of emodepside (10 μM). In the presence of low (52 nM) Ca^2+^, a situation in which voltage steps do not activate K^+^ currents, emodepside revealed a K^+^ current (Fig 8A, middle panel). However, in the presence of higher (290 nM) Ca^2+^, a situation in which the voltage steps generate typical outwardly rectifying K^+^ currents, emodepside inhibited the amplitude of the outward current (Fig 8B, bottom panel). We also noted, that in the presence of either 52 nM or 290 nM Ca^2+^, emodepside caused rapid membrane hyperpolarisation (n = 15; emodepside shifted the resting membrane potential from -55 ± 6 to -79 ± 3 mV, mean ± s.e.m.). During recordings made in current clamp mode we observed that a few minutes after emodepside application the membrane potentials reach steady-state values which were close to the K^+^ equilibrium potential (Em = -79.8 ± 6.5 mV, n = 15; E_K_ = -91.2 mV). Moreover, test pulses in the presence of emodepside for both 52 nM and 290 nM Ca^2+^ revealed linear current-voltage behaviour over the voltage range from -120 to + 60 mV in all experiments (n = 15; Fig 8A and 8B). Whilst activation and inhibition of the voltage-activated K^+^ current by emodepside was modified by the Ca^2+^ concentration, the emodepside-induced modification of the current-voltage relationship was largely independent of intracellular Ca^2+^ concentration. Thus in the presence of low intracellular Ca^2+^ (52 nM) where the Dm Slo channel was closed at most voltages and also at higher intracellular Ca^2+^ (e.g. 290nM) where the Dm Slo channel shows outward rectification, emodepside consistently induced a linear current-voltage behaviour of the Dm Slo channel (Fig 8B) indicating a reduction in the rectifying properties of the channel without any shift in reversal potential.

### Emodepside does not increase intracellular Ca^2+^ in HEK293 cells

**Fig 9:**
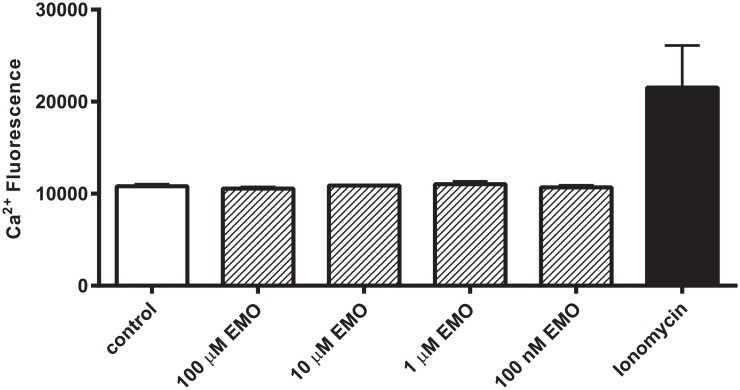
Emodepside does not increase cytosolic Ca^2+^ in HEK293 cells. Ca^2+^ fluorescence measured in HEK293 cells loaded with Fluo-3, AM dye after treatment with 1% DMSO (control), 100μM, 10μM, 1μM and 100nM emodepside (1% DMSO) or ionomycin (1% DMSO). The Ca^2+^ fluorescence was measured at excitation of 480p and emission of 525p in the FLUO Star OPTIMA machine. The readings for each treatment were taken in triplicate. Data were pooled from at least three independent experiments and are shown as mean ± s.e.mean.

It was important to substantiate that the actions of emodepside are mediated via a direct interaction with the channel rather than indirectly via an increase in intracellular Ca^2+^, possibly by acting as an ionophore [29]. In order to test this HEK293 cells were loaded with the Ca^2+^ fluorophore Fluo-3,AM and exposed to emodepside. Whilst a positive control, treating cells with ionomycin (10 μM), elicited a robust increase in Ca^2+^ fluorescence, no increase in intracellular Ca^2+^ was observed even at high micromolar concentrations of emodepside (Fig 9).

## Discussion

In this study we have investigated the cyclooctadepsipeptide compound emodepside as a novel modulator of the BK/SLO-1 Ca^2+^ -activated K^+^ channels from nematode, insect and human.

We established the requisite Ca^2+^ concentration for channel activation in order to establish experimental conditions in which to investigate the ability of emodepside to act as a channel modulator. The nematode Ca^2+^ -activated K^+^ channel differs from the insect and human channel in terms of its Ca^2+^ sensitivity. Thus we observed that 100μM Ca^2+^ was required to record Ce SLO-1 currents in contrast to 290–300 nM for Dm Slo and hum KCNMA1. This is consistent with the micromolar concentration of Ca^2+^ used to characterise the *C*. *elegans* SLO-1 channel in *Xenopus* oocyte expression studies [16] and patch clamp recordings from *C*. *elegans* body wall muscle which showed that 100μM internal Ca^2+^ concentration is required to observe SLO-1 channel openings [30]. The gating of the channel by Ca^2+^ is regulated by the gating ring [31] and thus these observations suggest functional differences between the nematode channel compared to human and insect in this region. The requirement of the nematode channel for high micromolar concentrations of Ca^2+^ for activation indicates that physiological regulation may be achieved only by close juxtaposition of the K^+^ channel with voltage-gated Ca^2+^ channels in the plasma membrane so that local, transient elevations in cytosolic Ca^2+^ may activate the channel as suggested by the Ca^2+^ domain hypothesis [32].

Emodepside showed efficacy at *C*. *elegans* SLO-1 and human KCNMA1 channels heterologously expressed in HEK293 cells. However, whilst emodepside affected both SLO-1 and KCNMA1 currents, there were distinct differences in the effect observed. Emodepside enhanced *C*. *elegans* SLO-1 currents by up to 80% and shifted the voltage-activation to a lower threshold. This resembles the effect of increased Ca^2+^ concentration on the voltage-activation range of BK channels [16,23,24,33]. In contrast, KCNMA1 currents were subject to biphasic modulation by emodepside, an initial transient facilitation followed by a more prolonged inhibition. Moreover, the maximal facilitation exerted by 100 nM emodepside was only 30%, considerably less than that observed for SLO-1. This difference in the efficacy of direct modulation of the nematode and the human channel by emodepside is consistent with the selective toxicity of the compound as an anthelmintic. There is corroboration for this from experiments in which either nematode *slo-1* or human *kcnma1* were expressed in *C*. *elegans slo-1* null mutants which were otherwise insensitive to emodepside. Whilst expression of nematode *slo-1* conferred sensitivity to emodepside on the *slo-1* null mutants [12,18], 10 to 100 fold higher concentrations of emodepside were required to impair mobility in transgenic lines expressing the human channel [18]. We also noted that the qualitative features of the impaired mobility caused by emodepside in the lines expressing the human channel were different from the effects seen in lines expressing *C*. *elegans slo-1*. In the latter a flaccid paralysis was observed when worms were exposed to low concentrations of emodepside and this effect was phenocopied by a *slo-1* gain of function mutant consistent with potent activation of the channel by emodepside [11]. In contrast, micromolar concentrations of emodepside applied to *C*. *elegans* expressing the human channel KCNMA1 impaired mobility but did not elicit a flaccid paralysis [18]. This led us to suggest that emodepside at micromolar concentrations is an inhibitor of the mammalian channel [18] and this is directly supported by the inhibition of KCNMA1 currents observed here. Whilst the inhibition of KCNMA1 by emodepside may be of relevance in predicting side-effects that might be encountered in its future use in tropical medicine.

The experiments with Dm Slo provide evidence for an additional effect of emodepside on Ca^2+^ -activated K^+^ channels, namely an ability to diminish the rectifying properties of the channel. This effect of emodepside on Dm Slo was independent of the intracellular Ca^2+^ concentration. Thus, in either low or high Ca^2+^, emodepside exposure led to the appearance of inward current i.e. there was a loss of the characteristic outward rectifying properties of the channel. It will be interesting to explore whether this phenomenon is observed in isoforms of slo channels from species of nematodes other than *C*. *elegans*.

The effect of emodepside on nematode muscle has been extensively investigated [4,9] and the observations made from muscle recordings are consistent with those from the recombinant expressed channels described here. Electrophysiological studies on the parasitic nematode *Ascaris suum* have shown that a cyclooctadepsipeptide compound, PF1022A, causes a Ca^2+^ and K^+^-dependent relaxation of body wall muscle [4]. Latterly it was shown that emodepside increases activation of voltage-dependent K^+^ currents in the muscle of *A*. *suum* [5], again consistent with the shift in voltage-activation seen in heterologously expressed *slo-1* observed in this study. BK channels, including *C*. *elegans* SLO-1, assemble with accessory subunits *in vivo* and it is possible these may impact on emodepside modulation [24] but the similar actions of emodepside on the heterologously expressed channels and currents recorded from native tissue argue that this is unlikely to play a significant role.

Our observations, and previous reports, indicate that the modulation of the Ca^2+^ -activated K^+^ channels in HEK293 cells by emodepside cannot be ascribed to an elevation of intracellular Ca^2+^. The parent compound for emodepside, the cyclooctadepsipeptide PF1022A, does not increase intracellular Ca^2+^ in CaCo-2 cells [34]. Here we conducted a similar study on HEK293 cells and showed that a supramaximal concentration of emodepside (100μM) had no significant effect on the intracellular concentration of Ca^2+^. There is evidence from *A*. *suum* that *in vivo* emodepside may act indirectly to modulate SLO-1 via an intracellular signalling cascade involving nitric oxide and protein kinase C [5]. However, given that we have shown that emodepside differentially modulates Ce SLO-1, Dm Slo and hum KCNMA1 expressed in HEK293 cells, this is an unlikely explanation for the modulation we have observed. Whilst it cannot be discounted that additional indirect mechanisms might operate *in vivo*, our data indicate that emodepside exerts a modulatory action on SLO-1 and KCNMA1 that is independent of a change in intracellular Ca^2+^ and likely reflects a direct modulation of the channel.

Taken together, our data substantiate a direct interaction of emodepside with *C*. *elegans slo-1* [19]. This first cross-phyla analysis of *C*. *elegans*, human and *Drosophila* slo/BK indicate a complex modulation of these Ca^2+^ -activated K^+^ channels by emodepside in which only the nematode channel exhibits a marked facilitation. This is underpinned by a shift in voltage-activation and provides molecular insight into its potency as an anthelmintic. The data reinforce the impetus to investigate the utility of this compound, or related drugs which target slo channels, for the treatment of human helminthiases. Recently it has been shown that there is a synergistic action of the filaricidal compound diethylcarbamazine with emodepside on *Ascaris suum* muscle slo currents and it would be interesting to investigate the mechanism of this in heterologously expressed slo channels [35]. From a broader perspective, the effects of emodepside reported here show that the cyclooctadepsipeptides provide a route to understanding a new pharmacophore harboured by BK/SLO-1 channels [13] which has important relevance for the therapeutic exploitation of this target.
